# Clinical and imaging analysis to evaluate the response of patients with anti-DPPX encephalitis to immunotherapy

**DOI:** 10.1186/s12883-022-02649-7

**Published:** 2022-04-05

**Authors:** Jun Xiao, Pei-cai Fu, Zhi-jun Li

**Affiliations:** Department of Neurology, Tongji Hospital, Tongji Medical College, Huazhong University of Science and Technology, Wuhan, 430030 People’s Republic of China

**Keywords:** Anti-DPPX encephalitis, Clinical and imaging spectrum, Immunotherapy

## Abstract

**Background:**

To report the main spectrum and new clinical and imaging characteristics of dipeptidyl-peptidase-like protein 6 (DPPX) antibody-associated encephalitis, and to evaluate the effect of immunotherapy.

**Methods:**

A retrospective analysis of nine patients with anti-DPPX encephalitis was performed, and all previously reported cases in the literature were reviewed. A cell-based indirect immunofluorescence assay using human embryonic kidney 293 cells transfected with DPPX was used.

**Results:**

Nine patients were identified (median age, 51 years; range, 14–65 years) with prodromal fever, diarrhea, or weight loss, followed by rapid progressive encephalopathy characterized by cognitive disorder. One patient who received methylprednisolone therapy and a trial of tacrolimus showed substantial improvement and had no relapse by the 6-month follow-up. Our comprehensive literature review demonstrated that 53 cases were reported, of which more than half had prodromal weight loss (52.8%) and gastrointestinal disorders (58.5%). Cognitive disorders (74.6%) and brainstem/spinal cord disorders (75.5%) were the most common major symptoms. A greater proportion of Chinese patients than non-Chinese patients had abnormalities on brain magnetic resonance imaging specific for encephalitis (70.0% vs. 23.3%, *P* < 0.001). Our study is the first to report three patients with anti-DPPX encephalitis who had sleep disorders with rapid eye movement sleep behavior disorder, limb paralysis (two), severe pleocytosis, elevated protein levels (two) in the cerebrospinal fluid, and increased T2/FLAIR signal abnormalities in the bilateral hippocampus, temporal lobe, amygdala, basal ganglia, thalamus, centrum semiovale, and frontal and parietal lobes in seven patients (77.8%).

**Conclusion:**

Our study expands the clinical and imaging phenotypes of anti-DPPX encephalitis. Further studies elucidating the entire clinical spectrum of anti-DPPX encephalitis, its pathogenic mechanisms, and prognosis under long-term immunosuppressive therapy are warranted.

**Supplementary Information:**

The online version contains supplementary material available at 10.1186/s12883-022-02649-7.

## Background

Autoimmune encephalitis, which is usually sensitive to immunotherapy, is a group of inflammatory diseases mediated by antibodies against the neuronal surface and synaptic proteins, receptors, and ion channels [1]. Newly identified antibodies for autoimmune encephalitis include metabotropic glutamate type 5 [2], gamma-aminobutyric acid receptor type A [3], and dipeptidyl peptidase-like protein 6 (DPPX) antibody [4].

DPPX, a cell surface regulatory subunit of the voltage-gated A-type Kv4.2 potassium channel, is expressed in neuronal somata and dendrites. It is localized to the hippocampus, cerebellum, and myenteric plexus [5, 6], and its related autoimmunity presents with a multiregional neurological phenotype. Anti-DPPX encephalitis is a rare type of autoimmune encephalitis, first described by Boronat et al. in 2013, and is caused by cell surface DPPX autoantigens [4]. Clinically, this disease is characterized by memory loss, agitation, confusion, psychiatric symptoms, seizures, tremors, myoclonus, ataxia, diarrhea, and weight loss [4, 7, 8]. Immunotherapy is widely used in the majority of patients with anti-DPPX encephalitis and may be beneficial to most patients regardless of the duration of symptoms. To date, only a few cases of anti-DPPX encephalitis have been reported, and little is known about the clinical spectrum and treatment outcomes of this disease. Thus, the diagnosis and treatment of anti-DPPX encephalitis remain challenging based on the variety of clinical symptoms and signs.

Here, we report the clinical features and imaging characteristics of nine patients with anti-DPPX encephalitis and investigate their responses and outcomes following treatment using several immunotherapy strategies. We also reviewed all previously reported cases to expand the clinical and imaging phenotypes, aiming to offer new recommendations for immunological treatment.

## Methods

### Patients

This study included nine patients admitted to the Department of Neurology, Tongji Hospital, Wuhan, China, from December 2018 to December 2019. Service-tested patient sera and cerebrospinal fluid (CSF) were found to be positive for anti-DPPX antibodies. Cranial magnetic resonance imaging (MRI) was performed in all patients. Clinical information was obtained from certified neurologists through a structured written questionnaire (Supplementary data). The patients’ clinical characteristics are described in detail below and summarized in Table 1.

**Table 1:** Demographic, clinical features, immunologic data and treatment of the patients with DPPX antibodies

	Patient 1	Patient 2	Patient 3^a^	Patient 4	Patient 5	Patient 6^b^	Patient 7	Patient 8	Patient 9
Demographic									
Sex	F	F	F	M	M	F	F	M	F
Age at onset, y	65	47	62	35	14	51	35	54	52
Months to onset	12	0.5	3	0.5	0.5	0.5	4	1	6
Clinical Features									
Prodromal symptoms	Headache	Fever, diarrhea	Weight loss (10 kg), diarrhea, headache;	Headache	Fever, gastroparesis and constipation	Fever, headache, weight loss (5 kg)	Vertigo, diarrhea	Headache	Fever
Main symptoms	Memory loss, executive dysfunction, delusions, hyperreflexia	Delirium in 11 days to cognitive disorder, aphasia, limb paralysis, RBD, vertigo	Bradykinesia, tremor, memory loss, RBD, hyperreflexia, nystagmus	Stiffness, sleep disorder (insomnia), generalized seizure	Confusion, cognitive disorder, limb paralysis, respiratory failure, hyperreflexia	Memory loss, insomnia, ataxia, nystagmus	Generalized seizure, insomnia, RBD	Somnolence, cognitive disorder, myoclonus, insomnia	Delirium, cognitive disorder, recurrent generalized seizures,
Brain MRI	Bilateral hippocampus T2/FLAIR increased signals	Bilateral temporal lobe and amygdala T2/FLAIR increased signals	Nonspecific white matter changes	Left hippocampus and basal ganglia T2/FLAIR increased signals	Extensive abnormal signals in the brainstem, basal ganglia and bilateral white matter	Extensive abnormal signals in the frontal lobe, basal ganglia, hippocampus and cerebellum	Normal	Nonspecific white matter changes	Bilateral temporal lobe, hippocampus and left frontal lobe T2/FLAIR increased signals
Immunologic data									
CSF	WBC: normal Protein: normal	WBC: normal Protein: normal	WBC: normal Protein: normal	December 2018 WBC: 370, Protein: 852 January 2019 WBC: 20, Protein 549	March 2019 WBC: 220, Protein:2096 May 2019 WBC: 10, Protein: 469	WBC: normal Protein: normal	WBC: normal Protein: normal	WBC: normal Protein: 958	November 2018 WBC (−), Protein: 609 December 2018 WBC (−), Protein (−)
DPPX antibody	Serum: 1:100 CSF: ND	Serum: 1:10 CSF: ND	Serum: 1:32 CSF: ND	Serum: 1:32 CSF: 1: 3.2	Serum: 1:320 CSF (−)	Serum: 1:100 CSF: 1:1	Serum: 1:10 CSF: (−)	Serum: 1:1000 CSF: 1:10	December 2018 Serum:1:10 CSF: (−) October 2019 Serum:1:32 CSF: ND
Treatment/response	IVMP, oral steroids/ marked improvement	IVMP, oral steroids/ marked improvement, but relapsed with steroid taper	Oral steroids/ marked improvement	IVMP, Tacrolimus/ mild, but steady improvement	IVMP, IVIg/ incomplete improvement	IVMP, Rituximab/ marked improvement	IVMP, oral steroids/ marked improvement	IVMP, oral steroids/ marked improvement	IVMP, oral steroids/ marked improvement, but relapsed with steroid taper

*Abbreviations*: *DPPX* dipeptidyl-peptidase-like protein-6, *ND* not done, *IVMP* intravenous methylprednisolone, *IVIg* intravenous immunoglobulins

^a^ Micropapillary carcinoma of thyroid was found during the course of the disease

^b^ Non-Hodgkin’s lymphoma was found during the examinations

This study was approved by the Ethics Committee of Tongji Hospital and written informed consent was obtained from all patients or their representatives before enrollment for the use of serum, CSF, and clinical information for research purposes.

### Serologic and CSF studies

Serum was obtained from all nine patients, and paired CSF specimens were obtained from six patients. Anti-DPPX antibodies were detected using a cell-based indirect immunofluorescence assay with human embryonic kidney 293 (HEK293) cells transfected with DPPX as previously reported [4]. Biochips (Euroimmun, Lübeck, Germany) presented the DPPX-antigen in fixed HEK 293 cells. HEK293 cells were transfected with plasmids containing pDNA3 and the human *DPPX* gene using Lipo2000 (Invitrogen, Carlsbad, CA, USA). After incubation for 2–3 days, the reactivity of the antibodies was detected using patient serum or CSF (dilutions were 1:40/1:2) using a fluorescein isothiocyanate-conjugated goat antibody (Sigma, St Louis, USA) according to the manufacturer’s instructions. The serum and/or CSF were tested for the following antibodies associated with paraneoplastic syndromes and autoimmune inflammatory disorders: N-methyl-D-aspartate (NMDA), alpha-amino-3-hydroxy-5-methylisoxazole-4-propionate1 (AMPA1), AMPA2, leucine-rich glioma-inactivated 1 (LGI1), γ-aminobutyric acid B (GABAB), contactin-associated protein-like 2 (Caspr2), IGLON5, aquaporin-4 (AQP-4), myelin oligodendrocyte glycoprotein (MOG), and glial fibrillary acidic protein (GFAP)-method: cell-based immunofluorescence assay (Euroimmun, Lübeck, Germany); Hu, Yo, Ri, Ma2, CV2 and Amphiphysin-method: EUROLINE PNS 12 Ag (Euroimmun, Lübeck, Germany).

### Review of reported patients with anti-DPPX antibodies

We reviewed all previous cases (44) reported until September 2020, where anti-DPPX antibodies were detected to evaluate the spectrum of symptoms, response to treatment, and imaging characteristics.

### Statistical analysis

All statistical analyses were performed using SPSS software 16.0. Continuous variables are reported as median (range, minimum–maximum) and were compared using the Mann–Whitney U test. Categorical variables are represented as percentages and frequencies and were compared using the chi-squared test or two-tailed Fisher’s exact test. Statistical significance was set at *P* < 0.05.

## Results

### Demographic and clinical features

The main clinical findings of the nine identified patients are summarized in Table 1. Six patients were women, and the median age at onset was 51 years (range, 14–65 years). Five patients had prodromal headache, four had fever, three had severe diarrhea, and two had weight loss. The development of neurological disorders progressed for a median of 1 month (insidious in four, subacute in three, and acute in two). Patients developed rapidly progressive encephalopathy characterized by cognitive disorder or memory loss (7), accompanied by sleep disorders, including insomnia, rapid eye movement sleep behavior disorder (RBD) (3), and limb paralysis (2). Examination revealed nystagmus (2) and hyperreflexia (3). Tumor screening detected B-cell non-Hodgkin’s lymphoma in one patient (case 6), and micropapillary carcinoma of the thyroid was found during the course of the disease in another patient (case 3).

**Fig. 1:**
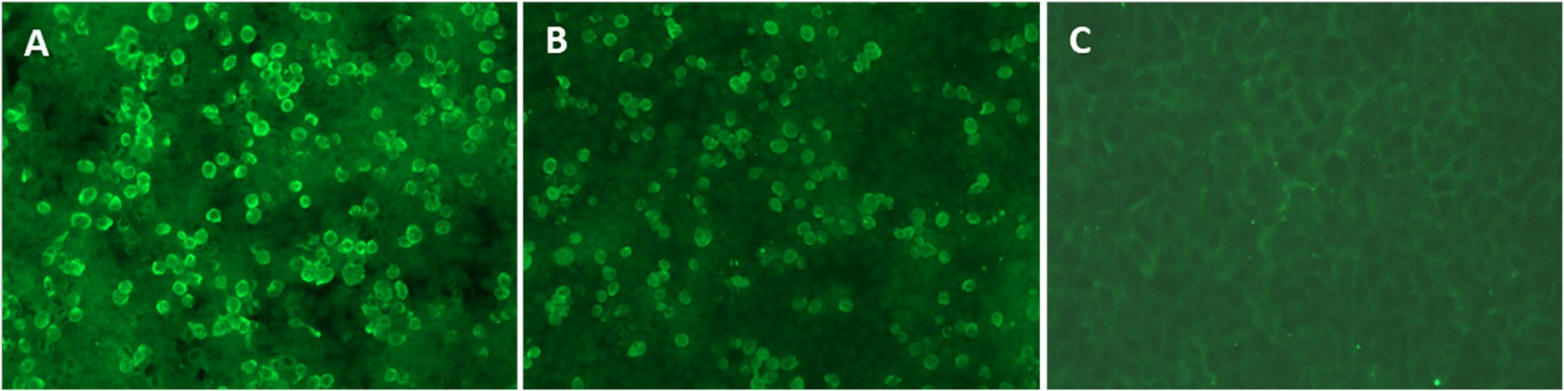
Immunofluorescence in serum and CSF in patient with anti-DPPX encephalitis. Positive reaction with transfected HEK 293 cells expressing DPPX with serum (**A**) (titer: 1: 1000); and with CSF (**B**) (titer: 1:10). (**C**) A negative control

Routine blood work results were normal in all nine patients. CSF data were available for all patients; white cell counts were elevated in two patients (370 and 220 × 10^6^/L, normal range, 0–8 × 10^6^/L) and protein levels were elevated in four patients (549 mg/L, 2096 mg/L, 958 mg/L, and 609 mg/L; normal range, 150–450 mg/L) without evidence of any infectious agents. As shown in Fig. 1, anti-DPPX antibodies were also detected by immunofluorescence in nine patients (nine serums and six CSF specimens). All patients showed anti-DPPX antibody seropositivity, whereas three CSF specimens were anti-DPPX antibody-positive (1:3.2, 1:10, and 1:1, respectively). Tests for other antibodies associated with paraneoplastic syndromes and autoimmune inflammatory disorders were negative in all patients, except for patient 5, where highly positive titers for autoantibodies against GFAP (serum: 1:320; CSF: 1:10) were noted.

### Responses to treatment

All patients in whom anti-DPPX antibodies were detected received multiple immunotherapies. Five patients received intravenous methylprednisolone (1 g for 3 days, 500 mg for 3 days, 240 mg for 3 days, and 120 mg for 3 days), followed by oral 80 mg prednisolone (slowly tapered over 6 months to 5 mg daily). All symptoms, including memory loss, tremor, and ataxia, showed marked improvement. However, as prednisolone was slowly tapered over 6 months to 5 mg daily, two patients experienced a relapse of symptoms (patient 9, serum titer of 1:10 before immunotherapy, serum titer of 1:32 at follow-up after 10 months). Only one patient received oral steroids owing to the presentation of mild symptoms that subsequently resolved, except for very slight ataxia during the heel-to-toe test. A single treatment with intravenous (IV) methylprednisolone and a course of IVIg (0.4 g/kg/dose × 5 days), however, produced only an incomplete effect. All but two patients (patients 4 and 6) showed marked improvement immediately after one course of high-dose IVMP. Patients 4 and 6 did not demonstrate satisfactory improvement after one course of IVMP treatment, which was followed by a trial of tacrolimus (3 mg, bid) or rituximab (two cycles every 3 months, 375 mg/m^2^), respectively, and the patients had substantial recovery after 6 months of follow-up.

### Brain MRI changes in patients with anti-DPPX antibodies

**Fig. 2:**
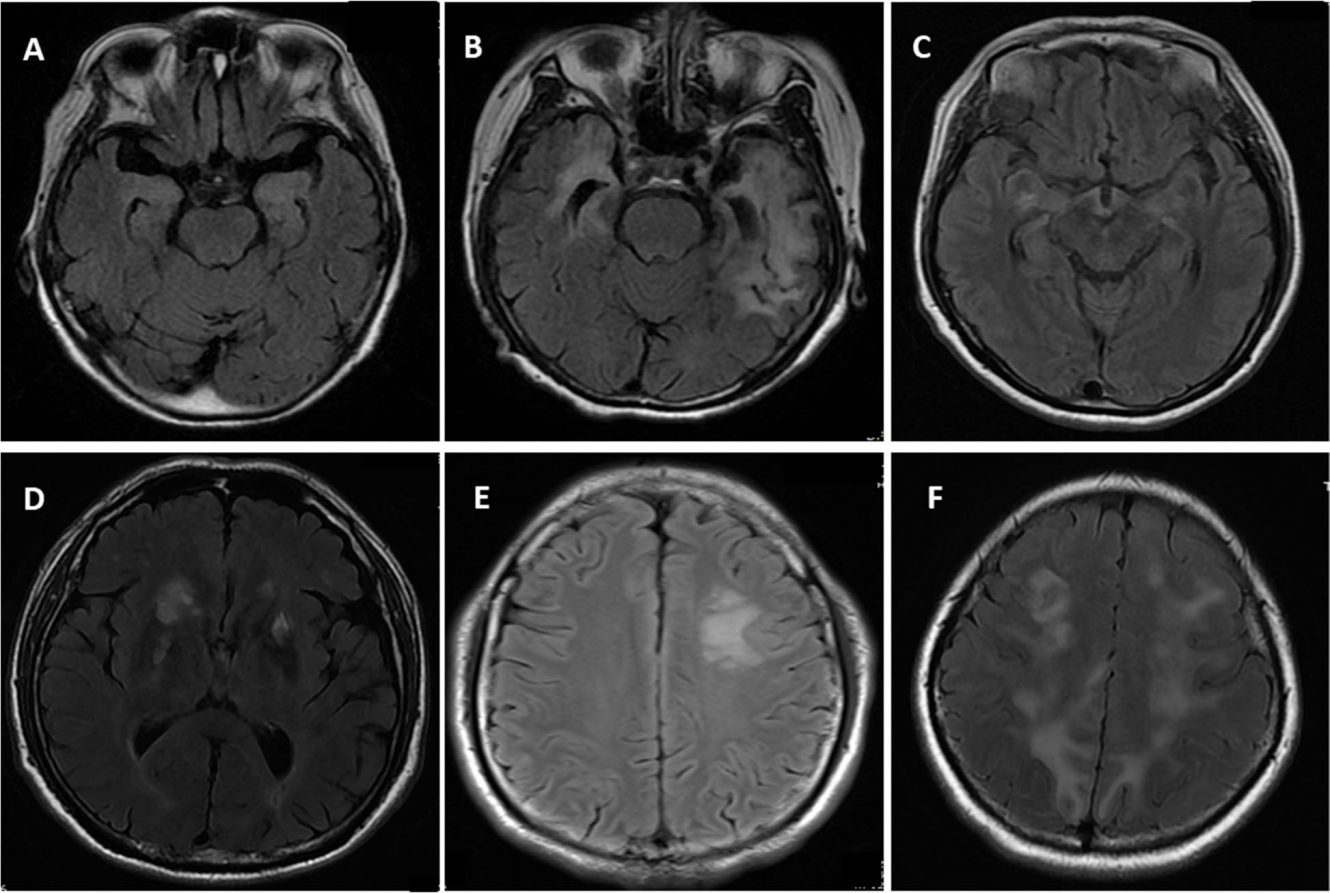
Brain T2/ FLAIR axial MRI specific changes of the patients with DPPX antibodies. **A** Mild increased T2/FLAIR signals of the bilateral hippocampus (Patient 1); **B** T2/FLAIR increased signals of the bilateral temporal lobe and hippocampus (Patient 9); **C** Mild T2/FLAIR increased signals of the bilateral amygdala (Patient 2); **D** Increased T2/FLAIR signals in the right basal ganglia and thalamus (Patient 7); E T2/FLAIR increased signals of the left centrum semiovale (Patient 4); F Extensive abnormal T2/FLAIR signals in the bilateral frontal lobe and parietal lobe (Patient 6)

Abnormalities were reported in the brain MRIs of eight patients, seven of whom had abnormalities specific to encephalitis. Only one patient showed nonspecific white matter changes. Figure 2A-C shows increased T2/FLAIR signals in the bilateral hippocampus, temporal lobe, and amygdala. T2/FLAIR signals in the right basal ganglia, thalamus, and left centrum semiovale and extensive abnormalities in the frontal and parietal lobes were observed (Fig. 2D-F). Changes in the functional cortical and basal ganglia regions mentioned above are very common in encephalitis, which could lead to symptoms such as cognitive dysfunction, ataxia, nystagmus, sleep disorders, mood disorders, autonomic symptoms, limb paralysis, and extrapyramidal symptoms.

### Summary of anti-DPPX encephalitis syndrome spectrum

From the discovery of anti-DPPX encephalitis until September 2020, 44 affected patients have been reported [4, 7–16]. Including our nine patients (a total of 53), the median age of all patients was 52 years (range, 13–76 years), and 34 (63%) were male. Only three patients were aged < 18 years (13, 14, and 15 years).

As shown in Table 2, prodromal headache occurred in six patients (11.3%), weight loss in 28 patients (52.8%), and gastrointestinal disorders (including diarrhea, gastroparesis, constipation, and abdominal pain) in 31 patients (58.5%). Cognitive disorders (74.6%) and brainstem/spinal cord disorders, including abnormal eye movement, dysphagia, stiffness, dysarthria, respiratory failure, vertigo, and hyperekplexia (75.5%), were the most common main symptoms of anti-DPPX encephalitis. Other common indications included myoclonus or tremors (49.1%), mood disorders (41.5%), sleep disorders (39.6%), dysautonomia (37.7%), cerebellar ataxia (34%), and psychosis (28.3%). Seven patients (12.2%) had generalized seizures and two (3.8%) had limb paralysis.

**Table 2:** Neurologic characteristics of 53 patients with DPPX antibodies

	No. (%)		
	Previous reported (*n* = 44)	Ours (*n* = 9)	Sum (*n* = 53)
Characteristics			
Cognitive disorders	32 (72.7)	7 (77.8)	39 (73.6)
Brainstem or spinal cord disorders^a^	36 (81.8)	4 (44.4)	40 (75.5)
Cerebellar ataxia	16 (36.4)	2 (22.2)	18 (34.0)
Myoclonus or tremor	23 (52.3)	3 (33.3)	26 (49.1)
Limb paralysis	0 (0)	2 (22.2)	2 (3.8)
Psychosis^b^	14 (31.8)	1 (11.1)	15 (28.3)
Headache	1 (2.3)	5 (55.6)	6 (11.3)
Weight loss	25 (56.8)	3 (33.3)	28 (52.8)
Sleep disorders^c^	17 (38.6)	4 (44.4)	21 (39.6)
Mood disorders^d^	19 (43.2)	3 (33.3)	22 (41.5)
Gastrointestinal symtoms^e^	27 (61.4)	4 (44.4)	31 (58.5)
Seizures	5 (11.4)	2 (22.2)	7 (12.2)
Dysautonomia^f^	17 (38.6)	3 (33.3)	20 (37.7)
CSF elevated white cell account	16/33 (48.5)	2 (22.2)	18/42 (42.9)
CSF elevated proteins	8/19 (42.1)	4 (44.4)	12/28 (42.9)
Brain MRI abnormalities	9 (20.5)	8 (88.9)	17 (32.1)
Brain MRI specific changes	1 (2.3)	7 (77.8)	8 (14.1)
Tumor Screening	5 (11.4)	2 (22.2)	7 (13.2)

*Abbreviations*: *DPPX* dipeptidyl-peptidase-like protein-6, *MRI* Magnetic Resonance Imaging

^a^ Abnormal eye movement; dysphagia; stiffness; dysarthria; respiratory failure; vertigo; hyperekplexia

^b^ Delirium; hallucination; delusion

^c^ Insomnia; hypersomnia; RBD

^d^ Depression; anxiety; apathy; appetite loss

^e^ Diarrhea; gastroparesis; constipation; abdominal pain

^f^ Diaphoresis; temperature dysregulation; urinary symptoms

Tumors were identified in eight patients (one with mantle cell lymphoma, four with B-cell non-Hodgkin lymphoma, one with B-cell chronic lymphocytic leukemia, one with breast adenocarcinoma, and one with micropapillary carcinoma of the thyroid).

Patients with anti-DPPX encephalitis might have elevated white cell counts and protein levels in the CSF (42.9%); however, no evidence of infectious agents was found. Tests for other antibodies associated with autoimmune inflammatory disorders were negative in all but two cases. In one patient, highly positive titers for autoantibodies against GFAP (serum: 1:320; CSF: 1:10) were detected, whereas AQP4 antibodies were found in the serum (1:375) but not in the CSF of the other patient [14].

Multiple immunotherapies were administered to 45 (81.1%) patients. Forty-seven patients underwent follow-up, including eight patients who did not receive immunotherapy; 23 (48.9%) had marked improvement, nine (19.1%) had incompletely resolved issues, nine (19.1%, including six treated without immunotherapy) had no improvement, and six (12.8%, including two without immunotherapy) patients died. Ten patients (21.3%) experienced clinical relapse, mostly during steroid tapering. Seven of these patients subsequently received immunosuppressants (rituximab, cyclophosphamide, or tacrolimus); all showed clinical improvement, whereas the other three patients had poor outcomes (two died, one had clinical progression).

Although not specified, we divided the reported cases into Chinese (*n* = 10) and non-Chinese (*n* = 43) subgroups according to the corresponding author’s address. As shown in Table S1, a greater proportion of Chinese patients than non-Chinese patients had abnormalities on brain MRI specific for encephalitis (70.0% vs. 23.3%, *P* < 0.001). No significant differences were found with regard to sex, median age at disease onset, characteristic clinical features, concurrent tumor, elevated CSF white cell counts and protein levels, percentage of positive oligoclonal bands and positive CSF DPPX antibody, non-specific brain MRI changes and response to acute-stage treatment, relapse or motality rate between the two subgroups.

## Discussion

DPPX, a widely expressed cell surface neuronal autoantigen, is a regulatory subunit of the voltage-gated Kv4.2 potassium channel complex [4, 17, 18]. DPPX proteins are distributed in both the nervous and enteric systems, including the hippocampus, cerebellum, cortex, brainstem, and submucous plexus neurons [4, 8, 17], which may lead to prominent manifestations, including encephalopathy, sleep disturbance, and gastrointestinal symptoms [18]. Although 44 cases of anti-DPPX encephalitis have been reported and studied, the clinical and imaging characteristics, mechanisms, and treatment strategies of this disease remain poorly understood. Here, we report nine new patients with anti-DPPX encephalitis and review all previously reported cases.

Anti-DPPX encephalitis is an extremely rare condition with a lack of classical radiological features. The diagnosis of anti-DPPX encephalitis remains challenging in clinical practice, and we tested for neural autoantibodies related to other more common types of autoimmune encephalitis, paraneoplastic syndromes, and anti-medullary diseases as a lab workup panel to rule out differential diagnoses, which increased the economic burden on the patients. As shown in Table 2, the clinical presentation of our cases was dominated by cognitive disorders, including memory loss (77.8%). The majority of patients had gastrointestinal symptoms (44.4%) and sleep disorders (44.4%), the features of which were similar to those previously reported (memory loss, 72.7%; gastrointestinal symptoms, 61.4%; sleep disorders, 38.6%). The atypical nature of clinical manifestations such as prodromal gastrointestinal symptoms and sleep disorders, along with encephalitis-related symptoms, such as memory loss, might be a clue for anti-DPPX encephalitis. Thus, we recommend routine screening for anti-DPPX antibodies for patients with the above suspicious clues.

With an increasing number of new patients, the spectrum of anti-DPPX encephalitis is likely to expand further. First, a noteworthy clinical feature was limb paralysis in two of our nine patients (22.2%), while the trunk and limbs were never severely involved in the previously reported 44 patients. Moreover, increasing evidence indicates a strong association between RBD and Parkinson’s disease, dementia with Lewy bodies (DLB), and voltage-gated potassium channel antibody-associated limbic encephalitis, principally against CASPR2 and LGI1 [19–22]. In our case series, we first reported three patients with anti-DPPX encephalitis who had a sleep disorder with features characteristic of RBD. Second, previously reported cases showed only mild pleocytosis (130 cells/μL, maximum) and elevated CSF protein levels (820 mg/L, maximum). In contrast, the changes were severe in our two cases (WBC count, 220 and 370 cells/μL; protein level, 2096 mg/L, maximum). Third, of the 44 previously reported patients, only nine had abnormalities on brain MRI; the changes were mostly nonspecific, including white matter changes and temporal lobe or hippocampal atrophy [23, 24]. Doherty suggested that FDG-PET showed increased FDG activity in the left medial, right lateral, and bilateral inferior recti, correlating with slow the phases of horizontal (LB) and vertical (UB) nystagmus [10]. Zhou et al. reported markedly decreased metabolic activity in the bilateral mesial temporal lobes (especially on the left side) on ^18^F-FDG PET-MR [13]. Increased T2/FLAIR signal abnormalities in the bilateral hippocampus, temporal lobe, amygdala, basal ganglia, thalamus, centrum semiovale, frontal lobe, and parietal lobe specific for encephalitis were observed in seven cases in our series (77.8%), which could explain the symptoms of limb paralysis. Meanwhile, in our pooled analysis, there was no statistical difference between the Chinese and non-Chinese patients, except for the percentage of specific brain MRI abnormalities, which were reported to occur more frequently in Chinese patients with anti-DPPX than in non-Chinese patients. However, the underlying reason remains unclear. These findings could enrich the understanding of the pattern of this relatively rare disease, enabling more comprehensive recognition of new cases. Therefore, in the context of an unclear etiology and various manifestations, anti-DPPX encephalitis should be considered.

Paraneoplastic and autoimmune encephalitis may occur with the use of antibodies against neural or glial antigens. Bien reported a case of paraneoplastic occurrence of antibodies against DPPX and AQP-4 in a patient with breast cancer and encephalitis [14]. Unlike the AQP-4 antibody, GFAP astrocytopathy is a meningoencephalomyelitis characterized by the detection of immunoglobulin G against GFAP in the CSF. Coexisting autoantibodies detected in the same patient, sometimes with a paraneoplastic cause, was called an overlapping syndrome [25–28]. We reported a 14-year-old patient with autoantibodies against both DPPX and GFAP; however, no tumor was detected within 1 year of follow-up. Therefore, further studies are required.

Patients with anti-DPPX encephalitis appear to respond well to early initiated immunotherapy, including IVMP or oral steroids, and relapse accompanies steroid tapering; therefore, long-term immunosuppressant therapy is needed to sustain optimal neurologic outcomes [4, 7, 8, 16]. Since consensus treatment guidelines for anti-DPPX encephalitis are not yet available, in our center, we developed a strategy based on the severity of the disease and tolerance of every single patient, which led to a wide range of steroid doses and treatment courses. Although rituximab alone or in combination with other therapies (cyclophosphamide or azathioprine) has been reported to be highly effective in numerous autoimmune disorders [29–31], as observed in our study and previous studies, its high costs and/or intolerable adverse events (deadly infections, granulocytopenia, and progressive multifocal leukoencephalopathy) interfere with its wide application.

Tacrolimus, a calcineurin inhibitor and first-line immunosuppressive drug, prevents organ and tissue rejection following transplantation [32–34] and promotes long-term safety and efficacy against myasthenia gravis [35] and neuromyelitis optica spectrum disorder [36]. Additionally, few reports have described tacrolimus therapy for autoimmune encephalitis, and we observed that most of the adverse events of tacrolimus were minor and well-managed [37]. Thus, we demonstrated the possibility of sequential steroid treatment (IVMP) with tacrolimus for anti-DPPX encephalitis. The patient had no relapse during the 6-month follow-up. Our research suggests that tacrolimus may be a promising immunosuppressant for anti-DPPX encephalitis. However, due to the relatively low incidence of this disease, there was only data from one patient; further observations and investigations are required to assess and confirm this beneficial effect.

## Conclusions

We conducted a retrospective study of patients with anti-DPPX encephalitis and reviewed previously reported cases. Our study expands the clinical and imaging phenotype of anti-DPPX encephalitis and reports symptoms of RBD and limb paralysis, severe pleocytosis, elevated protein levels in CSF, and specific abnormalities on cerebral MRI. We believe that our study significantly contributes to an improved understanding of anti-DPPX encephalitis, presents novel therapeutic strategies, and provides some clues for clinicians to recognize this rare condition to avoid misdiagnosis, decrease economic waste in unnecessary antibody tests, and encourage further in-depth mechanical studies. This study had some limitations. This was a single-center retrospective observational study, and because of the relatively low incidence of this disease, the molecular mechanisms underlying anti-DPPX encephalitis were not elucidated in our study. Furthermore, we consider it very important and necessary to discover a specific immunomodulator treatment strategy and to design a clinical trial for anti-DPPX encephalitis, but due to the neglect of a relatively large account of case reports and lack of in-depth mechanical studies, it is still challenging. Further studies elucidating the exact pathogenic roles and associated clinical spectrum are warranted to confirm the effect of immunosuppressive therapy.

## Supplementary Information

**Additional file 1: Table S1**. Comparison of demographic, clinical and neuroradiological characteristics between Chinese and non-Chinese subgroups.

## Data Availability

The datasets that support the findings of the study are available from the corresponding author upon reasonable request.

## Abbreviations

DPPX: Dipeptidyl-peptidase-like protein 6
RBD: Rapid eye movement sleep behavior disorder
CSF: Cerebrospinal fluid
MRI: Magnetic resonance imaging
HEK293: Human embryonic kidney 293
NMDA: N-methyl-D-aspartate
AMPA: alpha-amino-3-hydroxy-5-methylisoxazole-4-propionate
LGI1: leucine-rich glioma-inactivated 1
GABAB: γ-aminobutyric acid B
Caspr2: Contactin-associated protein-like 2
GFAP: Glial fibrillary acidic protein
IV: Intravenous
